# The Role of Heme Oxygenase-1 Promoter Polymorphisms in Perinatal Disease

**DOI:** 10.3390/ijerph18073520

**Published:** 2021-03-29

**Authors:** Ruka Nakasone, Mariko Ashina, Shinya Abe, Kenji Tanimura, Hans Van Rostenberghe, Kazumichi Fujioka

**Affiliations:** 1 Department of Pediatrics, Kobe University Graduate School of Medicine, 7-5-1 Kusunoki-cho, Chuo-ku, Kobe 650-0017, Japan; nakasone@med.kobe-u.ac.jp (R.N.); marikoa@med.kobe-u.ac.jp (M.A.); sabe@med.kobe-u.ac.jp (S.A.); 2 Department of Obstetrics and Gynecology, Kobe University Graduate School of Medicine, 7-5-1 Kusunoki-cho, Chuo-ku, Kobe 650-0017, Japan; taniken@med.kobe-u.ac.jp; 3 Department of Paediatrics, School of Medical Sciences, Universiti Sains Malaysia, Kubang Kerian 16150, Kelantan, Malaysia; hansvro@usm.my

**Keywords:** heme oxygenase-1, polymorphism, perinatal diseases, neonatal jaundice, genetics, SNPs, (GT)_n_ repeat

## Abstract

Heme oxygenase (HO) is the rate-limiting enzyme in the heme catabolic pathway, which degrades heme into equimolar amounts of carbon monoxide, free iron, and biliverdin. Its inducible isoform, HO-1, has multiple protective functions, including immune modulation and pregnancy maintenance, showing dynamic alteration during perinatal periods. As its contribution to the development of perinatal complications is speculated, two functional polymorphisms of the *HMOX1* gene, (GT)_n_ repeat polymorphism (rs3074372) and A(-413)T single nucleotide polymorphism (SNP) (rs2071746), were studied for their association with perinatal diseases. We systematically reviewed published evidence on *HMOX1* polymorphisms in perinatal diseases and clarified their possible significant contribution to neonatal jaundice development, presumably due to their direct effect of inducing HO enzymatic activity in the bilirubin-producing pathway. However, the role of these polymorphisms seems limited for other perinatal complications such as bronchopulmonary dysplasia. We speculate that this is because the antioxidant or anti-inflammatory effect is not directly mediated by HO but by its byproducts, resulting in a milder effect. For better understanding, subtyping each morbidity by the level of exposure to causative environmental factors, simultaneous analysis of both polymorphisms, and the unified definition of short and long alleles in (GT)_n_ repeats based on transcriptional capacity should be further investigated.

## 1. Introduction

Neonatal and perinatal complications have been recognized to occur in combination with genetic susceptibility and environmental factors such as maternal smoking, diabetes, or substance exposure. Several studies have demonstrated the importance of oxidative and inflammatory stressors for their essential roles in the development of diseases during the perinatal period. Thus, attempts to clarify the genetic components responsible for polymorphisms associated with the modulation of these stress cascades have been performed enthusiastically [1].

Heme oxygenase (HO) is the rate-limiting enzyme in the heme catabolic pathway, degrading heme into equimolar amounts of carbon monoxide (CO), free iron, and biliverdin, which is rapidly reduced to bilirubin [2]. Among the three identified isoforms of HO, inducible HO-1, also known as a stress-responsive protein, has been hypothesized to play a myriad of protective functions against several stressors, having antioxidant, anti-inflammatory, anti-apoptotic, anti-coagulation, anti-proliferative, and vasodilative properties [3,4,5,6]. In addition, HO-1 is also thought to play a role in maintaining immunologic balance in normal pregnancy, and its suboptimal expression is associated with a variety of perinatal complications [7]. 

Several researchers have reported that *HMOX1* gene expression is developmentally regulated. For example, its expression in the cerebral cortex is at its highest on day 1 after birth and gradually decreases until day 28 in mice [8]. However, lung HO-1 expression and HO activity peaked on gestational days e19.5 to e20.5, decreasing significantly from birth onwards in rats [9]. Intriguingly, intestinal HO activity varies with age and region, with HO activity increasing in an age-dependent manner up to six weeks after birth in the duodenum and jejunum, but in the distal ileum and colon, it is highest at one week and then significantly decreases [10]. These observations suggest the essential roles of the regulation of HO-1 in both normal and diseased states during perinatal periods. Thus, we hypothesized that HO-1 would significantly contribute to the development of perinatal diseases. 

The gene encoding HO-1 is located on chromosome 22q12, which consists of five exons and four introns. Among the reported polymorphisms in the *HMOX1* promoter regions, two polymorphisms have been identified as functional: a (GT)_n_ repeat dinucleotide length polymorphism (rs3074372) and A(-413)T single nucleotide polymorphism (SNP; rs2071746) [11]. Both have been shown to affect the transcriptional activity of *HMOX1* under several conditions [11,12,13,14]. For (GT)_n_ repeat dinucleotide length polymorphisms, the length of the (GT)_n_ microsatellite directly affects the level of gene transcription in vascular cells, with short repeats increasing the inducibility of HO-1. In an in vitro transient transfection assay, HO-1 transcriptional activity was upregulated by H_2_O_2_ exposure only in fusion genes with 16 and 20 repeats (short allele; S) but not with 29 or 38 repeats (long allele; L) [11]. Similarly, another in vitro assay with HL-1 atrial myocytes showed that the responsiveness of HO-1 transcriptional activity to tachypacing was inversely correlated with the length of the (GT)_n_ repeats (14 > 25 > 30 repeats). In atrial fibrillation patients, those homozygous for S (<27) alleles exhibited greater HO-1 expression in their atria tissues than those homozygous for L (≥27) alleles [15]. Recently, this investigation was confirmed for protein levels in the biologically pro-oxidant state, and the HO-1 protein level in the lymphocytes of patients with type 2 diabetes with the S/S genotype was three-fold higher than that in those with the L/L genotype [16]. 

The length of GT repeats is highly polymorphic, ranging from 16 to 45 based on several studies, and is known to have a bimodal distribution with peaks around 23 and 30 repeats in the general Caucasian, Hispanic, and Asian populations [15,17,18,19,20]. In the African population, especially in malaria-endemic areas, there are additional distinct peaks of around (GT)_39_ repeats, possibly due to the survival advantage from *Plasmodium falciparum* infection [21,22]. Walther et al. explained that the phenotypic severity of malaria in carriers of the S allele, which induces HO-1 expression, is protective to some extent; however, excessive HO-1 upregulation in response to inflammatory stress could also be deleterious [21].

The A allele of the A(-413)T SNP is also known to be associated with increased HO-1 promoter activity, as it is located in the *HMOX1* promoter region close to the (GT)_n_ repeat polymorphism. In a *Renilla* luciferase assay with bovine aortic endothelial cells, the A allele had significantly higher promoter activity than the T alleles in vitro [23]. Interestingly, the promoter activity of the A(-413)-(GT)_30_ allele was significantly higher than that of the T(-413)-(GT)_23_ allele, suggesting that A(-413)T SNP may be more responsible for activity [14]. However, this significant contribution of the A(-413)T SNP on transcriptional activity has been reported by only a single research group, and there have been conflicting results from in vitro functional studies, suggesting the necessity for further investigation [24].

These two polymorphisms have been widely studied with regard to the genetic susceptibility of human diseases, and significant associations have been reported in several adult diseases, especially malaria [21,22,25], cardiovascular diseases, atherosclerosis [14,26,27,28,29], chronic obstructive pulmonary diseases [11,30], certain cancers [31,32,33], and failure risks of organ transplantations [34,35,36]. Exner et al. clearly described and reviewed earlier work regarding the contribution of *HMOX1* polymorphism in adult diseases [4]. However, limited evidence is available for perinatal diseases. Thus, this article aimed to systematically review published evidence of the associations between *HMOX1* polymorphisms and the development of perinatal diseases and summarize them.

## 2. Materials and Methods

We performed a comprehensive literature search using PubMed with the keyword “Heme oxygenase-1 (*HMOX1*) polymorphism” with “neonate (neonatal, 20 hits)”, “pregnancy (8 hits)”, “maternal (8 hits)”, and “obstetrics (8 hits)”. We also collected the citations of the gathered articles, and 2 researchers (R.N. and K.F.) screened the related articles from 349 pieces of literature that matched with the single keyword, “Heme oxygenase-1 (*HMOX1*) polymorphism”. Then, we selected articles on perinatal diseases and categorized the selected articles into three groups: neonatal jaundice, bronchopulmonary dysplasia, and other perinatal morbidities, discussed below (Table 1).

## 3. Neonatal Jaundice

Neonatal jaundice is the most common cause of neonatal morbidity in the first week of life [52]. This is caused by an imbalance between bilirubin production and its elimination [53]. Severe neonatal jaundice, such as hemolytic jaundice, is associated with bilirubin-induced mortality or neurologic disorders. Today, the ethnic difference in susceptibility to neonatal jaundice is widely recognized, indicating a genetic contribution in addition to environmental factors. The relationship between genetics and neonatal jaundice is also implied by the results of twin studies, clinical significance of family history, and male–female difference [54]. To date, several genetic studies, including genome-wide association studies (GWAS), have disclosed the genetic polymorphisms associated with an increase in serum bilirubin levels, such as uridine diphosphoglucuronyltransferase (*UGT1A1*), glucose-6-phosphatedehydrogenase (*G6PD*), and solute carrier organic anion transporter family, member 1B3 (*SLCO1B3*) [55,56]. Since HO modulates the heme catabolic pathway and bilirubin production [2], the functional polymorphisms of *HMOX1* are a logical candidate for genetic susceptibility of neonatal jaundice. However, the *HMOX1* (GT)_n_ microsatellite polymorphisms cannot be determined using the GWAS approach. Thus, there is still no conclusive evidence regarding its relationship to neonatal jaundice development, and only a few studies with candidate gene approaches are available. In addition, a more straightforward positive contribution of short (GT)_n_ alleles on total serum bilirubin levels has been reported in healthy adults from the Indian and Uyghur populations [57,58].

In 200 Indian neonates, the (GT)_n_ repeat length ranged from 15 to 40 with a trimodal distribution, with a small peak at 15 GT repeats and larger peaks at 23 and 30 repeats. In this study, short (GT)_n_ repeats (<21) were independent risk factors for neonatal hyperbilirubinemia, despite no difference being noted in the prevalence of A(-413)T SNP between cases and controls. Intriguingly, all the carriers of 15 GT repeats, including the single allele carriers, were hyperbilirubinemic cases in this study [39]. A Turkish study with 152 term infants, which also had a bimodal (GT)_n_ peak at 23 and 30, ranging from 16 to 39, reported that patients with short repeats (<24 GT) had significantly higher prolonged unconjugated hyperbilirubinemia than healthy newborns [38]. In Japanese neonates, the (GT)_n_ repeat length ranged from 16 to 41, with a bimodal distribution peaking at 23 and 30 repeats. In this study, after excluding the *UGT1A1 G71R* polymorphism carriers, we found that carriers of the short allele (<22) were significantly associated with the development of neonatal hyperbilirubinemia [18]. In healthy Taiwanese newborns, whose gestational age was ≥ 35 weeks, the incidence of the short allele (<24) was significantly higher in infants with hyperbilirubinemia (Total Serum Bilirubin, TSB ≥ 15 mg/dL) than those without (59.0% (59/100) vs. 33.7% (116/344), *p* < 0.001, respectively) [43]. In another study on Japanese and German neonates, the (GT)_n_ repeat length ranged from 16 to 40, with a bimodal distribution peaking at 23 and 30 repeats. However, this study did not find a relationship between these polymorphisms and neonatal hyperbilirubinemia [37]. In Jewish neonates, the (GT)n repeat length ranged from 14 to 40, with a bimodal distribution peaking at 22 and 29 repeats. In this study, they found that the length of (GT)_n_ repeats did not affect the heme catabolism or total serum bilirubin values on the 3rd day in either G6PD-normal or deficient neonates [19]. In a recent, large study from China, the (GT)_n_ repeat polymorphisms did not affect the levels and changes in bilirubin during the first week of life [41]. Similarly, no association was found between the (GT)_n_ repeat polymorphisms and hyperbilirubinemia in breastfed, full-term Chinese infants [40]. Another study from China also reported no association between the (GT)_n_ repeat polymorphisms and neonatal hyperbilirubinemia [42].

In a prospective cohort study of African-American infants whose (GT)_n_ repeat polymorphisms were divided into three groups (short (S, <25), medium (M, 25–33), long (L, >33)), there was no significant difference found in bilirubin risk percentile based on the Bhutani nomogram between infants with at least one L allele, as opposed to at least one S allele (48.6 ± 34.0 vs. 44.9 ± 31.6; *p* = 0.51) [44].

Taken together, there is insufficient evidence regarding the genetic contribution of the A(-413)T SNP and several conflicting pieces of evidence of (GT)_n_ repeat polymorphisms in neonatal jaundice. Kaplan et al. likewise reported that there have been contradictory findings on the effects of *HMOX1* polymorphisms on neonatal jaundice, despite an increasing TSB trend in short (GT)_n_ repeats, possibly due to the influence of ethnic differences in each study. They also stated that the standardization of the definition of short (GT)_n_ repeats is required [59]. In addition, Zhou et al., in their meta-analysis, reported that newborns with short (GT)_n_ may be at an increased risk of neonatal hyperbilirubinemia; however, it is important to note that most of the studies included were from the Asian population [60].

Different categorizations of the (GT)_n_ repeat lengths made it difficult to interpret the results of each study comprehensively. However, most studies that categorized the S allele as ≤ GT_23_, the smaller of the bimodal peaks, showed a significant association, and those that adapted the categorization of a longer S allele did not. Taken together with the results of the in vitro transcription assay study, it seems that the carriers of substantially shorter (GT)_n_ repeats might be at increased risk of developing neonatal jaundice. This hypothesis was supported by a case report of massive hyperbilirubinemia (serum bilirubin > 70 mg/dL) in a boy with autoimmune hemolytic anemia who had both very short (GT)_n_ repeats ((GT)_21_/(GT)_22_). This may imply the augmenting effect of *HMOX1* promoter polymorphisms in bilirubin overproduction [61].

## 4. Bronchopulmonary Dysplasia (BPD)

BPD is the most common cause of morbidity and mortality in preterm newborns and is characterized by impaired alveolarization and vascularization in developing lungs [62]. Recently, genetic susceptibilities with higher estimated heritability for developing BPD have been implicated in preterm infants, as reported in a multicenter retrospective twin study [63], and many investigators are now attempting to identify the genetic polymorphisms responsible [64]. Multiple factors contributing to BPD, including infection or inflammation, oxidant stress, and ventilator-induced lung injury, have been determined [65,66]. HO-1 is implicated in antioxidant and anti-inflammatory defense mechanisms, as well as in angiogenesis and vasculogenesis [51,67], and is expressed abundantly in the human lung. In addition, *HMOX1* knockout causes enlarged alveolar spaces and increased lung apoptosis in BPD model mice induced by postnatal hyperoxia exposure, suggesting the regulation of postnatal lung repair after hyperoxia by HO-1 [68]. The large size of a (GT)_n_ repeat in the *HMOX1* gene promoter reduces HO-1 inducibility by reactive oxygen species in cigarette smoke, resulting in the development of chronic pulmonary emphysema, which has a pathophysiological background comparable to BPD [69]. Thus, *HMOX1* polymorphisms are one of the logical candidates for genetic susceptibility to BPD. Until now, there have only been two articles focusing on the relationships between *HMOX1* polymorphisms and the development of BPD.

One study from European groups with 342 preterm infants at ≤28 weeks gestational age reported no positive correlation between (GT)_n_ repeat polymorphism and BPD development [45]. Another study from an American group that included 117 preterm infants (birth weight <1200 g or ≤31 we gestation) reported no effect of both (GT)_n_ repeat and A(-413)T SNP on the development of BPD but a positive correlation between A(-413)T and the development of acute kidney injury [46].

Taken together, there has been no positive evidence about the contribution of *HMOX1* polymorphism on BPD, although the number of studies available is insufficient. However, since BPD has complicated pathogenesis mediated by multifactorial contributors, a detailed subgroup analysis would be necessary to clarify the true effect of these stress-protective gene polymorphisms. Now, we hypothesize that the interaction of *HMOX1* polymorphisms in BPD might be strengthened in the most at-risk populations with extensive oxidative stress, such as the presence of infection or long-term ventilator dependency. This agrees with the mechanisms of chronic obstructive pulmonary diseases or emphysema, which have a genetic predisposition associated with *HMOX1* polymorphisms that usually occur in smokers, as smoking is a known oxidative stressor [11,70]. 

## 5. Other Perinatal Morbidities

Since HO-1 has multiple protective functions via its bioactive products, several human neonatal diseases, tightly associated with oxidative stress or inflammation, are postulated to be modulated by its progression or severity. However, to date, only a few perinatal morbidities have been studied in association with *HMOX1* polymorphisms.

Spina bifida is a common congenital disorder characterized by abnormal closure of the embryonic neural tube. Its etiologies are multifactorial, including low maternal folate intake, maternal obesity, and maternal diabetes, each of which is associated with oxidative stress [17]. Since this etiology is also implicated in a strong genetic contribution, SNPs related to folate production or oxidative stress have been investigated. Recently, we carried out a case-control study with 300 Californian cohorts to investigate the association between two functional *HMOX1* polymorphisms and this entity. In this study, although we observed a doubling in risk for the A allele in A(-413)T among non-Hispanic whites, we did not find an obvious risk association in the whole population [17].

HO-1 also plays an important role in regulating vascular tone and blood pressure through CO production and is crucial during pregnancy to maintain the normal maternal vascular tone and fetal hemodynamic functions [71]. We have previously shown that the mouse model of partial HO-1 deficiency (*HMOX1* heterozygote knockout mouse, *HMOX1*^+/−^) caused morphological changes in the placenta and elevations in maternal blood pressure during pregnancy [72]. In addition, our previous data showed that partial deficiency of HO-1 might affect the infiltration and differentiation of lymphocytes in the placenta, resulting in an inadequate fetomaternal interface [73]. Currently, insufficient expression of HO-1 during pregnancy is regarded as a key etiology in the development of pregnancy complications, and a number of studies have been performed to clarify the function of HO-1 in preeclampsia or spontaneous miscarriage.

Preeclampsia is a syndrome of hypertension, proteinuria, and generalized vasoconstriction, which causes devastating maternal and fetal morbidities. The reduction of HO-1 expression in the placenta has been reported in cases of preeclampsia. A large Finnish case-control study examining the relationship between *HMOX1* polymorphism and this entity in 760 patients and 791 controls, in which allele frequencies of (GT)_n_ repeats are comparable to others, clarified an association between the long allele of (GT)_n_ repeats and non-severe, late-onset preeclampsia, but not severe preeclampsia [47]. The authors speculated that the effect of increased HO-1 expression via (GT)_n_ repeats might be too modest to modulate disease susceptibility in the most severe forms of the complication in which placental perfusion is severely impaired and stressed the importance of identifying the subtypes of these diseases. In a Brazilian case-control study examining plasma HO-1 levels and (GT)_n_ repeats in preeclampsia (n = 91) and healthy patients (n = 90) in 2019, there was no significant difference in plasma HO-1 levels and the LL or LS + SS allele frequency of (GT)_n_ repeats between the two groups [48]. The same authors reported another study that investigated the involvement of *HMOX1* polymorphism in the responsiveness to antihypertensive treatment in preeclampsia patients and revealed that the TT genotype of A(-413)T SNP was more frequent in methyldopa–non-responsive patients than in responsive patients (30% vs. 11%, *p* = 0.02) [49]. Likewise, in a large cohort study investigating the distribution of A(-413)T SNP in the *HMOX1* gene in preeclampsia patients and healthy women from China, no significant difference was found in the allele frequency between preeclampsia patients and healthy women or between patients with mild and severe preeclampsia [50].

Recurrent miscarriage is defined as three or more consecutive pregnancy losses before 20 weeks of pregnancy. Familial clustering and reduced placental HO-1 expression have been reported. In an investigation from a German Caucasian study with 169 idiopathic recurrent miscarriages and 129 control patients, the positive association between the carrier state of the S allele (S ≤ 27) and idiopathic recurrent miscarriage was disclosed; however, there was no significant difference with respect to S allele frequencies among the groups. A cautious interpretation of this result is warranted because the number of S/S genotypes is higher in the control group than in the idiopathic recurrent miscarriage group in this study [51].

## 6. Discussion

The contribution of *HMOX1* promoter polymorphisms to human diseases has been studied since the 1990s; however, evidence regarding perinatal disease is still insufficient. Several studies may have established the relationship between the extremely short allele of (GT)_n_ and neonatal jaundice; however, for other morbidities, no positive evidence can be obtained except for pregnancy complications. Regarding the occurrence of diseases, the importance of the synergistic contribution of genetic and environmental factors is well recognized. Regarding immunologic responses, for example, complicated cross-talk of genetics and environmental influences have been reported in the inhibitory repertoire of natural killer (NK) cells. While it is mainly controlled by genetics, environmental influences also play a role in determining the overall phenotype [74]. The patients studied during the series included highly environmentally heterogeneous background cases. Thus, to elucidate the true effect of these stress-responsive gene polymorphisms, we might have to focus on the very subtypes under significant stress circumstances. In terms of synergistic effects, the simultaneous analysis of the two *HMOX1* polymorphisms or haplotype analysis might be important, though not well investigated until now, because a modest increase in the disease risks in one polymorphism can be easily abolished by others with opposite functions. Further, the highly diverse definition of short and long alleles of GT repeats makes it difficult to compare the studies consistently, in addition to insufficient information about the transcriptional capacity of (GT)_n_ repeat polymorphisms. Thus, a more precise and stepwise genotype-expression correlation study is still highly mandatory to make sense of this controversial issue.

## 7. Conclusions

Several reports have shown the positive contribution of *HMOX1* polymorphisms to the development of neonatal jaundice; however, insufficient evidence is available regarding other perinatal complications. For better understanding, subtyping each morbidity by the level of exposure to causative environmental factors, simultaneous analysis of both polymorphisms, and the unified definition of short and long alleles in (GT)_n_ repeats based on transcriptional capacity should be further investigated.

## Figures and Tables

**Table 1:** Overview of the studies investigating the association between *HMOX1* polymorphisms and perinatal morbidities.

Disease	Author	Polymorphism	Sample Size	Ethnicity	(GT)_n_ Repeat Length Categorization	Polymorphism Associated with Disease
Neonatal Jaundice						
Hyperbilirubinemia needing phototherapy	Kanai 2003 [37]	(GT)_n_ repeat	211	Japanese German	S ≤ 26 M 27–32 L ≥ 33	No
Prolonged unconjugated hyperbilirubinemia	Bozkaya 2010 [38]	(GT)_n_ repeat	152	Turkish	S < 24 M 24–29 L ≥ 30	Yes
Hyperbilirubinemia during the first two weeks of life	Tiwari 2013 [39]	(GT)_n_ repeat -413 A/T	200	Indian	S < 21 L ≥ 21	Yes
TB values on the 3rd day of life	Kaplan 2014 [19]	(GT)_n_ repeat	168	Jewish	S ≤ 24 M 25–33 L ≥ 34	No
Hyperbilirubinemia on day 3 or later before discharge	Zhou 2014 [40]	(GT)_n_ repeat rs9607267 rs2071749	949	Chinese	S < 27 M 27–32 L ≥ 33	No
Bilirubin levels and changes during the 1st week of life	Zhou 2014 [41]	(GT)_n_ repeat	988	Chinese	S < 27 M 27–32 L ≥ 33	No
Hyperbilirubinemia requiring phototherapy	Yang 2015 [42]	(GT)_n_ repeat	237	Chinese	S ≤ 23 M 24–29 L ≥ 30	No
Hyperbilirubinemia in the 1st week of life	Katayama 2015 [18]	(GT)_n_ repeat	108	Japanese	S < 22 L ≥ 22	Yes
Hyperbilirubinemia during hospital course	Weng 2016 [43]	(GT)_n_ repeat	444	Taiwan	S < 24 L ≥ 24	Yes
Bilirubin risk percentile prior to discharge	Schutzman 2018 [44]	(GT)_n_ repeat	180	African-American	S < 25 M 25–33 L > 33	No
Bronchopulmonary Dysplasia						
BPD	Poggi 2015 [45]	(GT)_n_ repeat	342	Italian	S < 25 L ≥ 25	No
BPD	Askenazi 2015 [46]	(GT)_n_ repeat -413 A/T	117	American	S ≤ 27 L > 27	No
Others						
Spina Bifida	Fujioka 2015 [17]	(GT)_n_ repeat -413 A/T	300	American	S < 26 L ≥ 26	No
Non-severe and Late-onset Preeclampsia	Kaartokallio 2014 [47]	(GT)_n_ repeat	1538	Finnish	S ≤ 25 L > 25	Yes
Preeclampsia	Sandrim 2019 [48]	(GT)_n_ repeat	181	Brazilians	S ≤ 25 L > 25	No
Responsiveness to antihypertensive drugs in Preeclampsia	Sandrim 2020 [49]	-413 A/T	398	Brazilians	-	Yes
Preeclampsia	Xianping 2020 [50]	-413 A/T	2955	Chinese	-	No
Idiopathic recurrent miscarriage	Denschlag 2004 [51]	(GT)_n_ repeat	291	Caucasian	S ≤ 27 L > 28	Yes

BPD; bronchopulmonary dysplasia.

## Data Availability

The data presented in this study are available in the article.
